# Natural Products Isolated from Oriental Medicinal Herbs Inactivate Zika Virus

**DOI:** 10.3390/v11010049

**Published:** 2019-01-11

**Authors:** Mariana N. Batista, Ana Cláudia S. Braga, Guilherme Rodrigues Fernandes Campos, Marcos Michel Souza, Renata Prandini Adum de Matos, Tairine Zara Lopes, Natalia Maria Candido, Maria Leticia Duarte Lima, Francielly Cristina Machado, Stephane Tereza Queiroz de Andrade, Cíntia Bittar, Maurício L. Nogueira, Bruno M. Carneiro, Ricardo B. Mariutti, Raghuvir Krishnaswamy Arni, Marilia Freitas Calmon, Paula Rahal

**Affiliations:** 1 Laboratory of Genomic Studies, Sao Paulo State University—UNESP, São José do Rio Preto 15054-000, São Paulo, Brazil; batista_m.n@hotmail.com (M.N.B.); anabragga@gmail.com (A.C.S.B.); guilhermecampos07@gmail.com (G.R.F.C.); marcosmichel@gmail.com (M.M.S.); renatapram@hotmail.com (R.P.A.d.M.); tairinezlopes@gmail.com (T.Z.L.); naty_candido@ig.com.br (N.M.C.); mle.lima94@gmail.com (M.L.D.L.); francielly.cristinam@gmail.com (F.C.M.); teph94@gmail.com (S.T.Q.d.A.); cibittar@gmail.com (C.B.); brunocopo@yahoo.com.br (B.M.C.); rahalp@yahoo.com.br (P.R.); 2 Departamento de Doenças Infecciosas e Parasitárias, FAMERP, São José do Rio Preto 15090-000, São Paulo, Brazil; mnogueira@famerp.br; 3 Institute of Exact and Natural Science, Mato Grosso Federal University, Rondonópolis 78060-900, Mato Grosso, Brazil; 4 Department of Physics, Multiuser Center for Biomolecular Innovation, Sao Paulo State University-UNESP, São José do Rio Preto 15054-000, São Paulo, Brazil;ricardomariutti@yahoo.com.br (R.B.M.); arni@ibilce.unesp.br (R.K.A.)

**Keywords:** ZIKV, berberin, emodin, Vero E6 cells

## Abstract

Zika virus (ZIKV) has been associated with serious health conditions, and an intense search to discover different ways to prevent and treat ZIKV infection is underway. Berberine and emodin possess several pharmacological properties and have been shown to be particularly effective against the entry and replication of several viruses. We show that emodin and berberine trigger a virucidal effect on ZIKV. When the virus was exposed to 160 µM of berberine, a reduction of 77.6% in the infectivity was observed; when emodin was used (40 µM), this reduction was approximately 83.3%. Dynamic light scattering data showed that both compounds significantly reduce the hydrodynamic radius of virus particle in solution. We report here that berberine and emodin, two natural compounds, have strong virucidal effect in Zika virus.

## 1. Introduction

Zika virus (ZIKV), a member of the *Flaviviridae* family and the *Flavivirus* genus, is named after the forest where it was first identified in 1952 [1]. For approximately 60 years only a few minor epidemics have been identified, none of which had any major consequences. However, when this virus started to circulate in Brazil in 2015, a correlation was observed between ZIKV infection in pregnant women and poor fetal development, leading to severe conditions such as microcephaly and other neurological diseases [2]. Although the exact mechanism by which ZIKV triggers these diseases is still unclear, some relevant information is now available. For example, it is known that the virus can penetrate the placenta [3], infect progenitor neural cells, and disrupt development [4]. In addition to fetal disease, ZIKV has also been implicated in the development of the Guillain–Barré syndrome in adults [5].

Strategies to combat ZIKV infection include the development of vaccines [6] and the screening of molecules that inhibit the different phases of the viral lifecycle [7,8].

Berberine is an isoquinoline alkaloid belonging to the structural class of protoberberines and is encountered in many plants including *Berberis vulgaris* [9]. It exhibits several pharmacological properties and is particularly effective against entry and replication of many viruses, including the human cytomegalovirus (HCMV) [10], herpes simplex virus (HSV) [11], influenza virus [12], Chikungunya virus (CHIKV), and other *Alphaviruses* [13]. Emodin, an anthraquinone derivative, is also a naturally occurring compound derived from the Chinese herbs *Rheum palmatum* [14], *Polygonum multiflorum* [15], *Aloe vera* [16], and *Cassia obtusifolia* [17]. Emodin possesses a wide spectrum of pharmacological effects, including antiviral activity, against Coxsakie B5 virus (CVB5), human respiratory syncytial virus (HRSV) [18], influenza A virus [19], Epstein–Barr virus (EBV) [20], herpes simplex virus (HSV) [21], hepatitis B virus (HBV) [22], and Japanese encephalitis virus (JEV), which is another *Flavivirus* [23].

In this study, we tested the activity of the natural compounds berberine and emodin for their ability to inhibit ZIKV infection.

## 2. Materials and Methods

### 2.1. Emodin and Berberine

Stock solutions of Emodin and Berberine were diluted in 0.2% dimethyl sulfoxide (DMSO, Sigma-Aldrich, St. Louis, MO, USA). The working solutions were obtained by dilution, in different concentrations, of both drugs in Dulbecco’s modified Eagle’s medium (DMEM) (Sigma-Aldrich, St. Louis, MO, USA).

### 2.2. Cell Line

Vero E6 cells (ATCC CRL-1586^TM^), donated by Dr. Maurício Lacerda Nogueira, were cultured in DMEM (supplemented with 10% fetal bovine serum (Cutlab, Campinas, Brazil), 1%, 100 U/mL of penicillin, and 100 μg/mL of streptomycin (Invitrogen, New York, NY, USA) and maintained in a humidified 5% CO_2_ incubator at 37 °C.

### 2.3. Virus Preparation

To test the inhibitory potential of berberine and emodin, a Brazilian Zika virus strain isolated from a febrile patient in northeast Brazil [24] was used. An aliquot of this virus was inoculated onto *Aedes albopictus* mosquito cells, clone C6/36 (ATCC^®^ CRL-1660™), and incubated in Leibovitz’s L-15 medium (Cutlab, Campinas, Brazil) supplemented with 10% fetal bovine serum, 1%, 100 U/mL of penicillin, and 100 μg/mL of streptomycin for 10–14 days until the first cytopathic effects were observed. The same viral passage was used in all experiments.

### 2.4. Cytotoxicity of Berberine and Emodin in Vero E6 Cells

The cytotoxicity of berberine and emodin was evaluated in order to select the optimal non-toxic concentration of each compound in Vero E6 cells. For this assay, 5 × 10^3^ Vero E6 cells were seeded to each well of a 96-well plate and incubated for 24 h at 37 °C. Media was removed and replaced with DMEM containing different concentrations of berberine (0–475 µM) or emodin (0–80 µM). The effects of the compounds on the cells were analyzed at 24 h, 48 h and 72 h after treatment. The supernatants were removed, and a solution of 3-(4,5-dimethylthiazol-2-yl)–2,5-diphenyltetrazolium bromide (MTT; 1 mg/mL) (Sigma-Aldrich, St. Louis, MO, USA) was added to each well. The plates were then incubated for 30 min at 37 °C. Subsequent to incubation, formazan crystals were solubilized with 100 µL DMSO (Sigma-Aldrich, St. Louis, MO, USA), and the absorbance was measured at 570 nm.

### 2.5. Virucidal Effects of Berberine and Emodin

The ability of berberine and emodin to inhibit ZIKV in a pre-entry step, acting directly on the viral particle, was tested by a virucidal assay. Briefly, 2 × 10^5^ cells per well were seeded in a 12-well plate. Berberine or emodin were incubated at different concentrations (berberine: 20–160 µM and emodin: 0.04–40 µM) with ZIKV (10^6^ plaque-forming unit/ mL (PFU/mL)) for 1 h at 37 °C. A control, ZIKV incubated with the drug diluent (DMEM + DMSO 0.1%), was also performed. Subsequently, the drug-containing supernatant was titrated in Vero E6 cells and incubated for 96 h. Cells were fixed with 10% formaldehyde for 30 min before staining with a crystal violet ethanolic solution. The titer of the virus treated with different concentration of each compound was determined and compared to the control (diluent only).

### 2.6. Pre-Treatment of Vero E6 Cells with Berberine and Emodin

We also tested the influence of berberine and emodin on cell receptors involved in virus entry. For these experiment, one day prior to the assay, 2 × 10^5^ cells/well were seeded onto a 12-well. On the following day, the culture growth medium was replaced with DMEM containing 160 μM of berberine or 40 μM of emodin and incubation proceeded for 1 h at 37 °C. After one hour, the medium containing the compounds was removed, and the cell monolayer was washed thrice with PBS. The control was incubated only with DMEM + 0.1% DMSO (drug diluent). The treated cells were then infected with ZIKV and incubated at 37 °C for 1 h. The virus was then removed, culture media was added and cells were incubated for 96 h at 37 °C with 5% CO_2_. After fixation with 10% formaldehyde and staining with crystal violet, the number of foci was determined and compared to the non-treated control.

### 2.7. Selectivity Index (SI)

The half maximal inhibitory concentration (IC_50_) and half maximal cytotoxic concentration (CC_50_) of berberine and emodin were calculated using non-linear regression analysis. The IC_50_ and CC_50_ values, were used to calculate the selectivity index (SI) of each compound (SI = CC_50_/IC_50_). The SI suggests potential effectiveness, where the higher the value, the more promising the drug.

### 2.8. Analyses of Virions by Dynamic Light Scattering (DLS)

Dynamic Light Scattering (DLS) measurements of virions were carried out using freshly purified samples of ZIKV in polystyrene curettes with optical path lengths of 1 cm and at a concentration of 1.2 × 10^5^ PFU/mL in filtered PBS buffer (pH 7.5) after incubation for 1 h at 37 °C, both in the absence and presence of 40 µM emodin or 160 µM berberine. The results presented are the average values of 30 scans of 30 s, each obtained with a Zetasizer Nano S90 (Malvern, UK) equipped with a He-Ne laser (632.8 nm). The scattered light was detected at 90° and the experimental data were analyzed using the *Zetasizer* software.

### 2.9. Statistical Analysis

The results of viral inhibition were calculated as a percentage of the negative control (medium plus drug diluent). All statistical analyses were performed by one-way ANOVA with Tukey’s post-test using the GraphPad Prism 5.0 software (GraphPad Software, San Diego, CA, USA).

## 3. Results

### 3.1. Berberine and Emodin Presented Low Citotoxicity in Vero Cells

**Figure 1:**
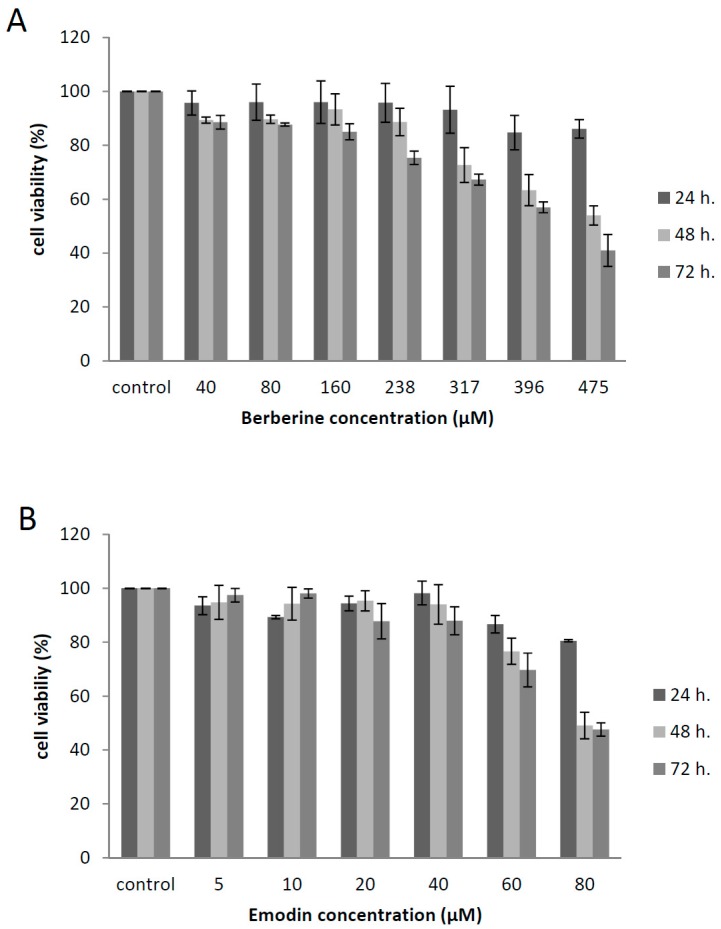
Viability of emodin- and berberine-treated Vero E6 cells. Cells were incubated with different concentrations of emodin (**A**) or berberine (**B**) and cell viability was evaluated after 24 h, 48 h, and 72 h.

We initially screened berberine and emodin for cellular cytotoxicity. The higher non-toxic concentrations were 160 µM for berberine and 40 µM for emodin (more than 80% cell viability) in all experimental times (Figure 1). By non-linear regression analysis, the CC_50_ values were calculated as 221 μM for berberine and 68.6 μM for emodin.

### 3.2. Berberine and Emodin Reduced the Infectivity of ZIKV in Vero Cells

**Figure 2:**
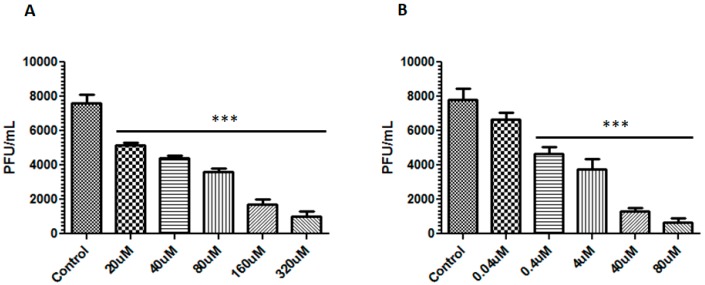
Virucidal assay for berberine and emodin. Different concentrations of berberine (**A**) and emodin (**B**) were incubated with Zika virus (ZIKV) (10^6^ PFU/mL) for 1 h. Cells treated with drug diluent was used as control. The results shown are the means (±SD) of three independent events, expressed as relative values compared to the untreated control. *** *p* < 0.0001 vs. control.

After determining the highest non-toxic concentration for each compound, a virucidal assay was performed to test the ability of these compounds to impair the virus particle infectivity. The supernatant containing infectious virus particles was incubated for 1 h at different concentrations of each compound (berberine: 20–160 µM and emodin: 0.04–40 µM) and subsequently used to infect Vero E6 cells. After 96 h, a considerable reduction of the foci numbers was observed for both compounds in a dose-dependent manner. When the viral particles were incubated with 160 µM of berberine, plaques reduced from 7.6 × 10^3^ PFU/mL (control) to 1.7 × 10^3^ PFU/mL, a reduction of 77.6% in ZIKV infectivity. When the particles were exposed to 40 µM of emodin, this reduction was approximately 83.3% (from 7.8 × 10^3^ PFU/mL to 1.3 × 10^3^ PFU/mL) (Figure 2). The IC_50_ values for berberine and emodin were 39.06 μM and 3.2 μM respectively, leading to a SI of 5.65 for berberine and 21.44 for emodin.

Although at reduced concentrations, berberine had a lower ability to inhibit the virus, all tested concentrations of berberine presented a significant reduction of foci number compared to the control (*p* < 0.001) (Figure 2A). Emodin inhibited the virus with a significant reduction of foci number for all tested concentrations (*p* < 0.001), except for 0.04 µM (Figure 2B).

### 3.3. Emodin Reduced ZIKV Entry in Vero Cells

**Figure 3:**
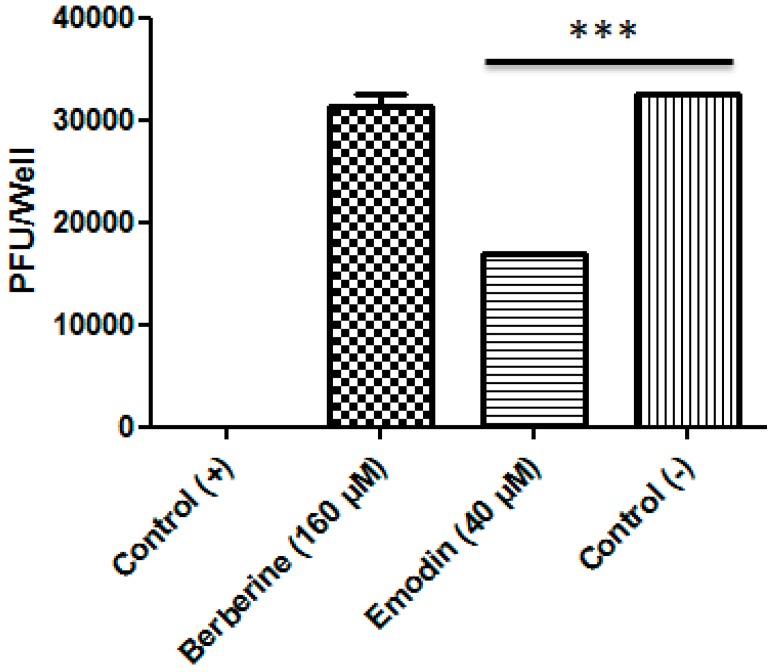
Pre-treatment of cells with emodin and berberine. Graphic of relative foci number after treatment of Vero E6 cells with berberine or emodin. Cells treated with drug diluent were used as negative control. Results are the means (±SD) from three independent experiments and are expressed as relative values compared to untreated cells. *** *p* < 0.0001 vs. control.

Finally, we tested the compounds as a pre-treatment, exposing the cells to each compound prior to infection. Vero E6 cells were incubated with berberine or emodin for 1 h at the highest non-toxic concentrations prior to ZIKV infection. Pre-treatment with berberine had no effect on virus entry. On the other hand, emodin reduced entry by 42.3% (plaques reduced from 3 × 10^4^ PFU/mL (control) to 1.73 × 10^4^ PFU/mL) (Figure 3).

### 3.4. Berberine and Emodin Impair ZIKV Particles

**Figure 4:**
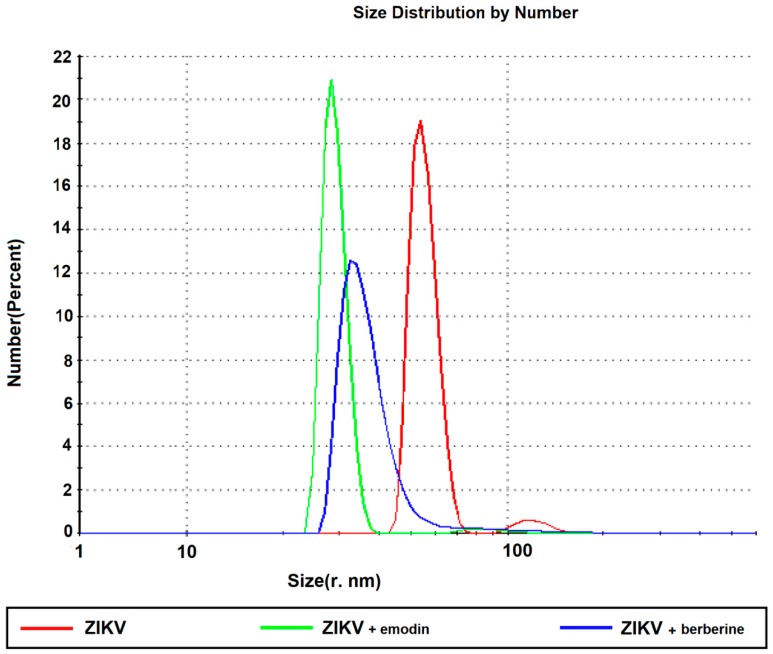
Dynamic light scattering analyses of virus particles after incubation at 37 °C in absence of drug (red line), in the presence of 40 µM emodin (blue line) and 160 µM berberine (green line), indicating the estimated hydrodynamic radius.

The hydrodynamic radius of ZIKV was determined both before and after incubation with berberine or emodin. The purified ZIKV particles are monodisperse (red line, Figure 4) with a correlation coefficient close to unity (Supplementary Figure S1) and the addition of 40 µM of emodin (green line, Figure 4) or 160 µM berberine (blue, Figure 4) led to a significant reduction of the hydrodynamic radius of the samples. ZIKV incubated with emodin presented a sharper profile than when incubated with berberine. Since the drugs were dissolved in DMSO, DLS measurements were also conducted with DMSO at the same concentration as the diluent, and did not indicate any significant difference with the native virus in a PBS buffer.

## 4. Discussion

Berberine and emodin, when evaluated in vitro, have virucidal effects on Zika virus. Berberine reduced virus infectivity by almost 80% in Vero E6 cells. This compound is present in two herbs widely used in natural therapies, *Coptis* sp. and *Berberis* sp. [9], and has been shown to be effective in controlling glycemia and lipid metabolism [25]. From an antimicrobial point of view, berberine inhibited many viruses, in particular influenza virus [12], CHIKV [13], and HSVs [11]. In these studies, berberine was effective at later stages of the viral cycle, such as during uncoating and replication. However, it should be noted that the viruses mentioned are not *Flavivirus.*

Emodin is an anthraquinone derivative present in several types of herbs and used primarily in Eastern medicine for the treatment of various health conditions [26]. This compound has also been shown to inhibit several viruses using different mechanisms; for example, emodin can block the coronavirus channel protein and inhibit the process of viral particle release from cells [27]. This compound also blocked the entry step of coxsackieviruses [28] and HSV [29]. We also observed a modest (42.31%) effect of emodin as a pre-treatment, therefore interfering in virus entry. However, the mechanism of action is not clear. In previous studies, the effect of emodin on virus entry was determined through its high affinity for the envelope phospholipid bilayer, and this interaction can lead to the rupture and destruction of the HSV-1 and VHSV viral particles [30,31]. A wide spectrum of biological activities has been described for emodin through different mechanisms of action [27,28,29,32,33] and among these, some are directly related to induced changes of the viral envelopes, such as 95% disruption of the herpes simplex virus type 1 (HSV-1) envelope [30]. The interaction of emodin with phospholipids and its effects in the integrity of vesicles and lipid extracts from *Escherichia coli* through the perturbation of the physical properties of the bilayer have been reported [31,34], supporting the direct effect of this anthraquinone on biological membranes. Berberine has also been described to act directly on biomembranes [35] or by inducing the increase of membrane permeability [36].

DLS measurements indicated differences in the estimated hydrodynamic radii of the virion samples before and after incubation with emodin or berberine. The calculated hydrodynamic diameter is indicative of the apparent size of the dynamic hydrated/solvated particle including the hydration sphere which encapsulates the virus particle. Thus, the observed change in the apparent hydrodynamic radius of the infectious viral particle upon addition of emodin and berberine could indicate disorder or disruption of the bilayer, or alterations in the solvation characteristics of the viral particles in solution.

Compounds that act inhibiting virus before the attachment to the host cell, such as berberine and emodin, can be used prophylactically at low concentrations since the number of infectious particles in a primary infection is relatively low. In addition, berberine can also spread to various organs of the body, such as the liver, kidneys, muscles, and brain, and remain there for a considerable period of time [37]. This is a desired characteristic for an antiviral targeting Zika virus when maintained at low concentrations, since many cell lines and tissues has been shown to be permissive to this virus [38,39,40]. Thus, infection via mosquito bites or through sexual contact could be controlled. Another beneficial feature of berberine is that it has low toxicity and no serious adverse effects in humans [41,42,43]. Some applications of berberine and emodin have encountered a few obstacles in pharmaceutical development, such as poor aqueous solubility [44,45] and rapid metabolism [46,47]. Recently, several nanoparticulate delivery systems for berberine and emodin have been reported, which have attempted to address the major pharmaceutical concerns associated with its systemic administration, with lipid-based nanocarriers being the most investigated [48,49,50,51,52,53,54,55].

## 5. Conclusions

Zika virus has drawn much attention within the scientific community since 2015 as a result of the outbreak in Brazil. Efforts have been made to identify effective drugs to treat this virus and to elucidate its fundamental characteristics. In this study, we showed that berberine and emodin, two natural compounds, have strong virucidal effect in vitro, directly impairing the ZIKV particles. After additional studies, given the high cellular viability in the active concentrations both compounds are good candidates for treatment of Zika virus-infected patients and possibly for preventive treatment in low doses in endemic areas.

## Supplementary Materials

Supplementary materials can be found at http://www.mdpi.com/1999-4915/11/1/49/s1.
