# Supplementary material for: Natural Products Isolated from Oriental Medicinal Herbs Inactivate Zika Virus
Source: Viruses. 2019 Jan 11;11(1):49. doi: 10.3390/v11010049 (PMC6356660; doi:10.3390/v11010049)
Supplement: Supplementary file 1 [file viruses-11-00049-s001.pdf]

Natural products isolated from oriental medicinal herbs inhibit Zika virus

Mariana N. Batista[a*], Ana Cláudia S. Braga[a*], Guilherme Rodrigues Fernandes Campos[a], Marcos Michel Souza[a], Renata Prandini Adum de Matos[a], Tairine Zara Lopes[a], Natalia Maria Candido[a], Maria Leticia Duarte Lima[a], Francielly Cristina Machado[a] Stephane Tereza Queiroz de Andrade[a], Cíntia Bittar[a], Maurício L. Nogueira[b], Ricardo B. Mariutti[d], Raghuvir Krishnaswamy Arni[d], Bruno M. Carneiro[a,c], Marilia Freitas Calmon[a&], Paula Rahal[a&]

[a] Laboratory of Genomic Studies, Sao Paulo State University - UNESP, São José do Rio Preto, São Paulo, 15054-000, Brazil; [b] Departamento de Doenças Infecciosas e Parasitárias, FAMERP, São José do Rio Preto, São Paulo, 15090-000, Brazil; [c] Institute of Exact and Natural Science, Mato Grosso Federal University, Rondonópolis, Mato Grosso, Brazil;

correspondence: macal131@gmail.com

[d] Department of Physics, Multiuser Center for Biomolecular Innovation, UNESP, São José do Rio Preto, SP, Brazil.

*The authors contributed equally to this work.

&The authors contributed equally to this work.

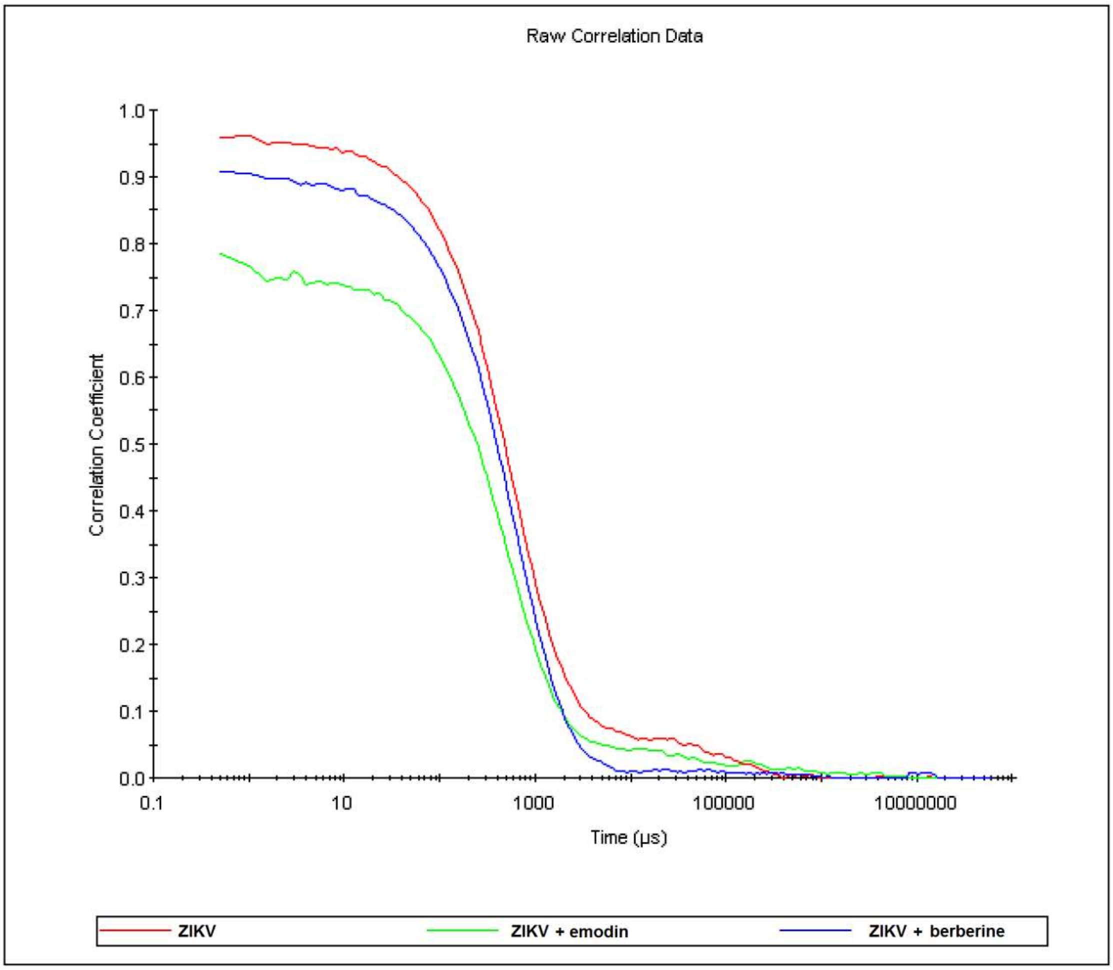

Supplementary Figure 1. DLS measured correlation curves of infectious virus particles after incubation at at 37°C in absence of drug (red line), in the presence of 40 μM emodin(blue line) and 160 μM berberine (green).
